# Genetic and Structural Basis for Selection of a Ubiquitous T Cell Receptor Deployed in Epstein-Barr Virus Infection

**DOI:** 10.1371/journal.ppat.1001198

**Published:** 2010-11-18

**Authors:** John J. Miles, Anna M. Bulek, David K. Cole, Emma Gostick, Andrea J. A. Schauenburg, Garry Dolton, Vanessa Venturi, Miles P. Davenport, Mai Ping Tan, Scott R. Burrows, Linda Wooldridge, David A. Price, Pierre J. Rizkallah, Andrew K. Sewell

**Affiliations:** 1 Department of Infection, Immunity and Biochemistry, Henry Wellcome Building, Cardiff University School of Medicine, Heath Park, Cardiff, United Kingdom; 2 Cellular Immunology Laboratory, Queensland Institute of Medical Research, Brisbane, Australia; 3 Computational Biology Unit, Centre for Vascular Research, University of New South Wales, Kensington, Australia; 4 Complex Systems in Biology Group, Centre for Vascular Research, University of New South Wales, Kensington, Australia; University of Birmingham, United Kingdom

## Abstract

Despite the ∼10^18^ αβ T cell receptor (TCR) structures that can be randomly manufactured by the human thymus, some surface more frequently than others. The pinnacles of this distortion are *public* TCRs, which exhibit amino acid-identical structures across different individuals. Public TCRs are thought to result from both recombinatorial bias and antigen-driven selection, but the mechanisms that underlie inter-individual TCR sharing are still largely theoretical. To examine this phenomenon at the atomic level, we solved the co-complex structure of one of the most widespread and numerically frequent public TCRs in the human population. The archetypal AS01 public TCR recognizes an immunodominant BMLF1 peptide, derived from the ubiquitous Epstein-Barr virus, bound to HLA-A*0201. The AS01 TCR was observed to dock in a diagonal fashion, grasping the solvent exposed peptide crest with two sets of complementarity-determining region (CDR) loops, and was fastened to the peptide and HLA-A*0201 platform with residue sets found only within TCR genes biased in the public response. Computer simulations of a random V(D)J recombination process demonstrated that both TCRα and TCRβ amino acid sequences could be manufactured easily, thereby explaining the prevalence of this receptor across different individuals. Interestingly, the AS01 TCR was encoded largely by germline DNA, indicating that the TCR loci already comprise gene segments that specifically recognize this ancient pathogen. Such pattern recognition receptor-like traits within the αβ TCR system further blur the boundaries between the adaptive and innate immune systems.

## Author Summary

The human immune recombination machinery can generate approximately 10^18^ unique αβ T cell receptor structures. The recombination event, once thought to be random, has now been shown to involve enzymatic biases during chromosomal rearrangement; additional biases occur during thymic selection and antigen-driven expansion in the periphery. The furthest extremes of these collective biases result in *public* T cell receptors (TCRs), defined as residue-identical receptors found across different individuals who share a common major histocompatibility complex (MHC) allele. One of the most prominent public T cell responses found in humans is raised against the GLCTLVAML (GLC) peptide from Epstein-Barr virus. We, and others, have previously shown that a public TCR constructed from the *TRBV20-1/TRBJ1-2* and *TRAV5/TRAJ31* gene segments dominates the GLC-specific repertoire. Here, we investigate the genetic, biophysical and structural forces that drive this public receptor, designated AS01, with *in silico* estimates of relative production frequencies during gene recombination, thermodynamic scanning and crystallographic studies of the AS01-GLC-A2 complex. We find that the TCRα and TCRβ amino acid sequences of AS01 are produced efficiently by a process of convergent recombination and employ unique residues, encoded only by the above-mentioned genes, to engage antigen in a highly specific manner.

## Introduction

Epstein-Barr virus (EBV), also called human herpesvirus 4 (HHV-4), is a genetically stable agent that has slowly co-evolved with our species and its antecedents for millions of years. EBV is typically transmitted orally during childhood, propagates in B cells and epithelia, and is shed for the lifetime of the host. More than 90% of the world's population is infected with EBV. This mutual coexistence is not without heavy resource cost for the host. Large populations of CD8^+^ αβ T lymphocytes are deployed for the purposes of EBV surveillance and suppression. These populations peak during asymptomatic primary infection [1], acute infectious mononucleosis (AIM) [2] and old age [3]. Across the entire EBV proteome, one of the most immunogenic CD8^+^ T cell targets is the HLA-A*0201-restricted GLCTLVAML peptide derived from the BMLF1 protein (residues 280–288; herein referred to as GLC-A2). During primary infection, up to 11% of the total peripheral CD8^+^ T cell pool can be specific for GLC-A2 [4]; this response contracts to 0.5–2.2% of the peripheral CD8^+^ T cell pool during persistent infection [4], but can swell again to 10% in old age [3]. Given the high *in vivo* frequencies of this response and the ubiquity of both EBV infection and the HLA-A*0201 allele, it is unsurprising that GLC-A2 is one of the most studied HLA class-I target antigens. Interestingly, initial investigations into the clonotypic nature of the GLC-A2 response revealed that CD8^+^ T cells are deployed with a biased T cell receptor (TCR) repertoire [5], [6], [7], [8] that is stable over time [9].

The TCR is a clonotypic, membrane-bound receptor that binds peptide-MHC (pMHC). Genetically, TCRs are rearranged into α- and β-chains from a selection of 176 variable (*V*), diversity (*D*), joining (*J*), and constant (*C*) genes on chromosomes 7 and 14. Random recombination of these genes generates only 5–10% of the potential diversity within the TCR repertoire; exonucleolytic activity, random N nucleotide additions at the V(D)J junctions [10] and αβ chain pairing contribute the remainder. Theoretical TCR diversity in humans has been placed in the region of 10^15^–10^20^ unique structures [11], [12], [13], with direct *in vivo* estimates greater than 2.5×10^7^ unique structures [14]. Structurally, TCR α- and β-chains fold to expose six highly flexible complementary determining region (CDR) loops that can contact the pMHC binding face. The germline-encoded CDR1 and CDR2 loops, from the *TRAV* and *TRBV* genes, participate heavily in MHC contacts and occasionally peptide contacts. The variable CDR3 loops, which span the V(D)J joints, are key to TCR diversity and participate heavily in peptide contacts. TCRs dock with pMHC complexes in a roughly diagonal fashion, such that the CDR3α loops are placed over the peptide N-terminus and the CDR3β loops lie over the peptide C-terminus.

In spite of the universe of TCR options available to the immune system, some pMHC antigens provoke the emergence of biased and predictable repertoires (reviewed in [15], [16]). Accordingly, the CD8^+^ T cell response to the GLC-A2 antigen is seen to provoke type III and type IV TCR bias. Type III bias is defined by memory T cells bearing identical TCR receptor protein sequences, often encoded by redundant codons, found between individuals presenting a common pMHC antigen. Type IV bias is defined by memory T cells bearing near identical TCR receptor protein sequences, differing by only one or two residues in the CDR3 loop, found between individuals presenting a common pMHC antigen. GLC-A2-specific responses exhibit biased usage of the *TRBV20-1*, *TRBJ1-2*, *TRAV5*, and *TRAJ31* genes and conserved CDR3 amino acid usage and length. We recently undertook a large scale *ex vivo* TCR sequencing analysis (754 transcripts), as well as a meta-analysis, of the GLC-A2 response and found that the most frequently shared (public) receptor comprised the above genes with the CDR3α and CDR3β core sequences CAEDNNARLMF and CSARDGTGNGYTF, respectively [17].

In order to gain insight into the structural basis underlying the emergence of this ubiquitous αβ receptor, we solved the structure of an archetypal GLC-specific TCR, derived from the CD8^+^ T cell clone AS01, in complex with the GLC-A2 antigen. In parallel, we performed a detailed thermodynamic dissection of the complex and identified key TCR contact hotspots via a biophysical mutagenesis scan. To investigate the genetic basis behind the dominant selection of the receptor, we performed computer simulations of a random V(D)J recombination process to assess the ease and frequency of manufacture. Herein, we describe the structural and genetic basis for the dominance of the AS01 TCR in EBV-infected humans.

## Results

### Overview of AS01-GLC-A2 complex

**Figure 1:**
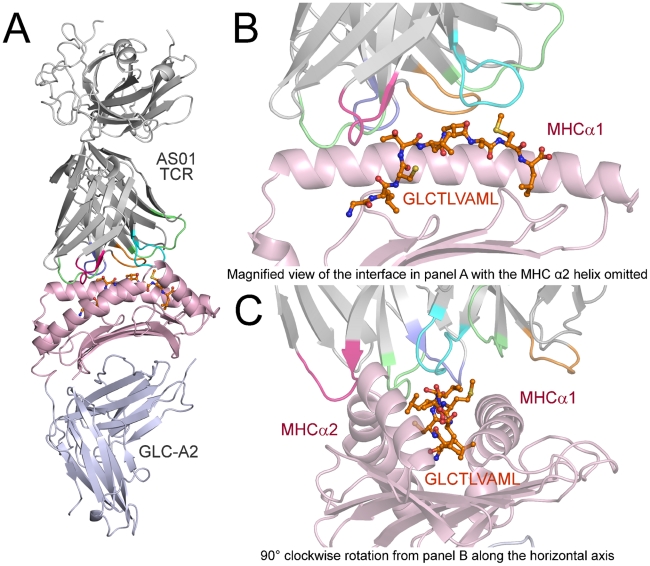
Overview of the AS01-GLC-A2 complex structure. (A) Ribbon representation of the AS01-GLC-A2 complex. The TCR α-chain and TCR β-chain are depicted in light grey; CDR1α, CDR2α, CDR3α, CDR1β, CDR2β and CDR3β are depicted in green, red, blue, lime green, orange and aqua, respectively. The HLA-A*0201 platform is depicted in light pink, with the stalk and β2-microglobulin in silver. The GLCTLVAML peptide is colored orange and represented in ball-and-stick format. (B) Magnified view of the AS01-GLC-A2 complex interface from the same angle as in panel A. The position of the AS01 TCR CDR loops over the central peptide bulge can be observed. For clarity, the MHC α-2 helix is omitted. (C) Magnified view of the AS01-GLC-A2 complex interface at 90° clockwise rotation from panel B along the horizontal axis. The overall position of the AS01 TCR CDR loops over both the MHC helices and the peptide can be observed.

**Figure 2:**
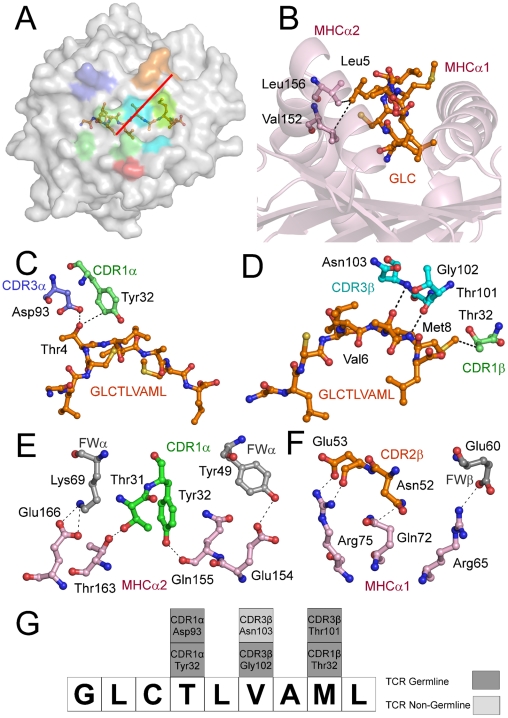
Interactions of the AS01-GLC-A2 complex. (A) Contact footprint of the AS01 TCR on the GLC-A2 surface. The HLA-A*0201 molecule is surface represented and colored grey; the GLCTLVAML peptide is shown in ball-and-stick format and colored orange. The binding footprint over the GLC-A2 surface of the AS01 TCR CDR1α, CDR2α, CDR3α, CDR1β, CDR2β and CDR3β loops are depicted in green, red, blue, lime green, orange and aqua, respectively. The TCR/pMHC crossing angle is depicted in red. (B) Interactions governing anchoring of Leu5 to the MHC α-2 helix. The MHC cleft is represented in ribbon format and the GLCTLVAML peptide, as well as the corresponding α-2 helix contacts, are represented in ball-and-stick format. (C) Antigen-specific interactions between the AS01 TCR CDR1α loop (green sticks), the CDR3α loop (blue sticks) and the N-terminus of the GLCTLVAML peptide. (D) Antigen-specific interactions between the AS01 TCR CDR1β loop (lime green sticks), the CDR3β loop (aqua sticks) and the C-terminus of the GLCTLVAML peptide. (E) MHC (light pink sticks) interactions with the genetically unique components of the TCR α-chain framework (FW) region (grey sticks) and CDR1 loop (green sticks). (F) MHC (light pink sticks) interactions with the genetically unique components of the TCR β-chain FW region (grey sticks) and CDR2 loop (orange sticks). (G) Schematic representation of TCR and contacts (hydrogen bond, salt bridge or van der Waals interactions) with peptide and the germline origins of the TCR contact residues. Only wholly non-germline encoded residues are depicted in light grey. Asp93 is partially encoded by non-germline DNA.

The structure of the AS01-GLC-A2 complex was determined to a resolution of 2.54 Å (Table 1). The final model had Rfree of 30.7% and Rcryst of 21.8%. The ratio, Rcryst/Rfree, falls within the accepted limits shown in the theoretically expected distribution [18]. The AS01 TCR was centrally perched on the GLC-A2 molecule over the exposed residues of the GLC peptide (Figure 1). As illustrated in Figure 2A, AS01 bound in a canonically diagonal fashion and was observed to dock at an angle of 41.7°, as calculated by the proposed TCR/pMHC crossing angle standard [19]. This crossing angle falls within the range of previous human TCR/pMHC class-I (pMHC-I) complexes (34°–80°, average 52.5°). The central residues of the GLC peptide bulged out from the MHC surface in the classical fashion. Unusually, however, Leu at residue P5, typically one of the most exposed regions of pMHC-I 9-mer peptides, was pulled down into the MHC cleft, kinking the backbone and making the adjacent residues, Thr at P4 and Val at P6, more solvent exposed (Figure 2B). The CDR1α/CDR3α loops and CDR1β/CDR3β loops were positioned on either side of the exposed peptide residues at P4 and P6, clasping each side of the peptide bulge (Figure 2C and 2D). The CDRα and CDRβ loops, as well as framework (FW) residues, shared in fastening the AS01 TCR to the MHC α-1 and α-2 helices (Figure 2E and 2F). A total of 20 contacts were made at the TCR/pMHC interface, comprising 4 peptide contacts and 16 MHC contacts (Table 2). This is the second lowest number of contacts observed across all human TCR/pMHC complexes to date [19], [20], [21], [22]. However, the total buried surface area (BSA) of the AS01-GLC-A2 complex was 2134 Å^2^, which falls within the normal range of previous human TCR/pMHC-I complexes (1471–2452 Å^2^, mean 1992 Å^2^) (Table 3; Figure 2A).

**Table 1:** Data collection and refinement statistics.

Parameters	Value
Data set statistics^a^	
Space Group	P2_1_2_1_2
Unit Cell parameters (Å)	a = 94.1, b = 122.5, c = 82.4
Radiation Source	DIAMOND I03
Wavelength (Å)	0.9763
Resolution (Å)	2.54 (2.61 – 2.54)
Reflection observed	251,677 (17,505)
Unique reflections	31,817 (2,222)
Completeness (%)	98.3 (93.4)
Multiplicity	7.9 (7.9)
I/Sigma(I)	12.2 (2.7)
Rmerge (%)	16.3 (83.2)
Refinement statistics^a^	
Measured Resolution Range (Å)	61.25 – 2.54
No reflections used	30,170 (2,105)
No reflections in Rfree set	1,603 (112)
Rcryst (no cutoff) (%)	21.8
Rfree (%)	30.7
Root mean square deviation from ideal geometry^b^	
Bond lengths (Å)	0.014 (0.021)
Bond Angles (°)	1.572 (1.936)
Mean residual B value after TLS (Å^2^)	14.866
Wilson B-factor (Å^2^)	43.1
Overall coordinate error (Å)	0.306

a Values in parentheses are for the highest resolution shell.

b Values in parentheses are target values.

N.B. One crystal was used for the full data set.

**Table 2:** Contacts between AS01 TCR and GLC-A2.

TCR region	TCR residue	MHC residue	Bond type^a^	Distance
CDR1α	Thr31 OG1	Thr163 OG1	HB	2.81
	Try32 OH	Gln155 O	HB	3.12
FWα	Tyr49 OH	Glu154 OE1	HB	3.16
	Lys69 NZ	Glu166 OE1	SB	3.15
	Lys69 NZ	Glu166 OE2	SB	3.04
CDR3α	Asn95 ND2	Gly62 CA	vdW	3.39
	Arg97 NH1	Glu60 OE1	SB	2.66
	Arg97 NH1	Arg65 NH1	vdW	3.26
	Arg97 NH1	Arg65 NH2	vdW	3.36
	Arg97 NH1	Arg65 CZ	vdW	3.28
CDR2β	Asn52 ND2	Gln72 CD	vdW	3.28
	Glu53 OE1	Arg75 NH1	SB	2.51
	Glu53 O	Arg75 NH2	HB	2.48
FWβ	Glu60 OE2	Arg65 NH1	SB	3.10
CDR3β	Asn103 ND2	Ala150 O	HB	2.94
	Asn103 ND2	Gln155 OE1	HB	2.65
	TCR residue	Peptide residue		
CDR1α	Tyr32 CE2	Thr4 OG1	vdW	3.36
CDR3α	Asp93 OD1	Thr4 OG1	HB	2.74
CDR1β	Thr32 CG2	Met8CE	vdW	3.33
	Asn103 N	Val6 O	HB	3.00

a Bond type defined as a hydrogen bond (HB), salt bridge (SB) or van der Waals (vdW).

**Table 3:** Analysis of human TCR/pMHC-I complexes.

TCR	PDB	MHC	Peptide	BSA^a^	Affinity^b^	SC total^c^	SC TCR/MHC^d^	SC TCR/Peptide^e^	Peptide contacts^f^	Reference
B7	1BD2	A*0201	LLFGYPVYV	1697	NT	0.643	0.597	0.798	60	[75]
A6	1AO7	A*0201	LLFGYPVYV	1816	2.0	0.632	0.642	0.640	47	[75]
JM22	1OGA	A*0201	GILGFVFTL	1471	5.6	0.635	0.698	0.610	29	[48]
LC13	1MI5	B*0801	FLRGRAYGL	2020	10.0	0.612	0.599	0.659	28	[47]
1G4	2BNR	A*0201	SLLMWITQC	1916	13.3	0.717	0.604	0.837	110	[76]
SB27	2AK4	B*3508	LPEPLPQGQLTAY	1752	9.9	0.715	0.594	0.825	65	[23]
DM1	3DX8	B*4405	EENLLDFVRF	2200	NT	0.595	0.536	0.763	37	[22]
MEL5	3HG1	A*0201	ELAGIGILTV	2452	18.0	0.625	0.575	0.763	29	[21]
ELS4	2NX5	B*3501	EPLPQGQLTAY	2400	NT	0.686	0.647	0.768	39	[77]
RA15	3GSN	A*0201	NLVPMVATV	2200	6.3	0.631	0.607	0.732	5	[20]
AS01	3O4L	A*0201	GLCTLVAML	2134	8.1	0.640	0.676	0.577	4	-

a Buried surface area (Å^2^) at the TCR/pMHC interface.

b Binding affinity (K_D_) in µM.

c Total shape complementarity index between the TCR and pMHC-I molecule.

d Shape complementarity index between the TCR and MHC-I molecule.

e Shape complementarity index between the TCR and peptide.

f Total number of hydrogen bonds, salt bridges and van der Waals force contacts between TCR and peptide within 3.4Å.

NT: Not tested.

Note: BSA, K_D_, and peptide contacts previously published in the final table column reference as well as [19].

### Structural basis underlying AS01 selection

The CD8^+^ T cell response to the GLC-A2 antigen exhibits Type III and Type IV bias *in vivo*, with identical or near identical TCRs within and between individuals [6], [7], [15], [16], [17]. The AS01-GLC-A2 structure provides insight into the selection of this public TCR. First, selection of the *TRAV5* gene can be accounted for by the presence of the Thr31-Try32 residue pair at the tip of the CDR1α loop (Figure 2E). These residues fix AS01 to the MHC α-2 helix via hydrogen bonds with Thr163 and with the mobile ‘gatekeeper’ Gln155 [23]. Engagement of the MHC α-2 helix is further strengthened through FW residues within the *TRAV5* gene-encoded chain. Tyr49 and Lys69 can be seen to form a hydrogen bond and salt bridge with Glu154 and Glu166, respectively (Figure 2E). In addition, Tyr32 in the CDR1α loop also assists in peptide recognition through a van der Waals interaction with Thr4 (Figure 2C). Of the 47 *TRAV* genes available for recombination, only the *TRAV5* gene encodes a Thr-Tyr pair in the CDR1α loop and only *TRAV5* encodes Tyr49 and Lys69 within the FWα region. The germline origins of these peptide contacts, as well as all TCR-peptide contacts, are shown in Figure 2G. Second, selection of *TRAJ31* over 56 other *TRAJ* gene options can be accounted for by the presence of Arg97 and the Asn94-Asn95 pair within the CDR3α core. The two Asn residues form a structural arch that stabilizes the CDR3α loop above the first peptide backbone hump residue at P4. Arg97 acts as a “hook” to stabilize the arch in a raised manner above Thr4. The raised CDR3α lip allows Asp93 to lie precisely level with Thr4 to form a hydrogen bond (Figure 2C). Only the *TRAJ31* gene encodes the Asn94, Asn95 and Arg97 pattern in the joining segment. Third, selection of *TRBV20-1* over the other 53 *TRBV* genes can be accounted for by gene-specific interactions. Asn52 and Glu53 at the tip of the CDR2β loop engage Gln72 and Arg75 of the MHC α-1 helix, respectively, through a van der Waals interaction, a hydrogen bond and a salt bridge (Figure 2F). Glu60, in the FWβ region, forms a salt bridge with Arg65 (Figure 2F). A peptide-specific interaction is achieved via Thr32 in the CDR1β loop, which engages Met8 through a van der Waals bond (Figure 2D). Genetically, a small number of *TRBV* genes encode an Asn-Glu pair in the CDR2β but only *TRBV20-1* encodes both the CDR2β Asn-Glu pair as well as FWβ Glu60 and CDR1β Thr32. Fourth, the importance of the contribution of the *TRBD1* gene is highlighted by the contacts made through Thr101, which is *TRBD1*-encoded. Thr101 makes a hydrogen bond with Met8 of the peptide (Figure 2D). Finally, selection of *TRBJ1-2* over the other 12 *TRBJ* gene options can be accounted for by the Tyr105-Thr106 pairing in the CDR3β loop terminus. Tyr105 forms a hydrogen bond with Arg98, found at the beginning of the CDR3β sequence. This internal brace stabilizes the entire loop structure. It is of particular interest that only the *TRBV20-1* gene encodes Arg at this position. Thr106 also has an important internal structural function, stabilizing the β-barrel formation through a methyl interaction with Phe29 of the CDR1β. It is of additional note that only *TRBV20-1* encodes Phe at this position when compared to all available 54 CDR1β loops. To conclude, only *TRBJ1-2* encodes a Tyr-Thr pair within the joining gene area, underlying its genetic preference associated with *TRBV20-1* in the public receptor.

### Binding affinity and thermodynamics of the AS01-GLC-A2 complex

**Figure 3:**
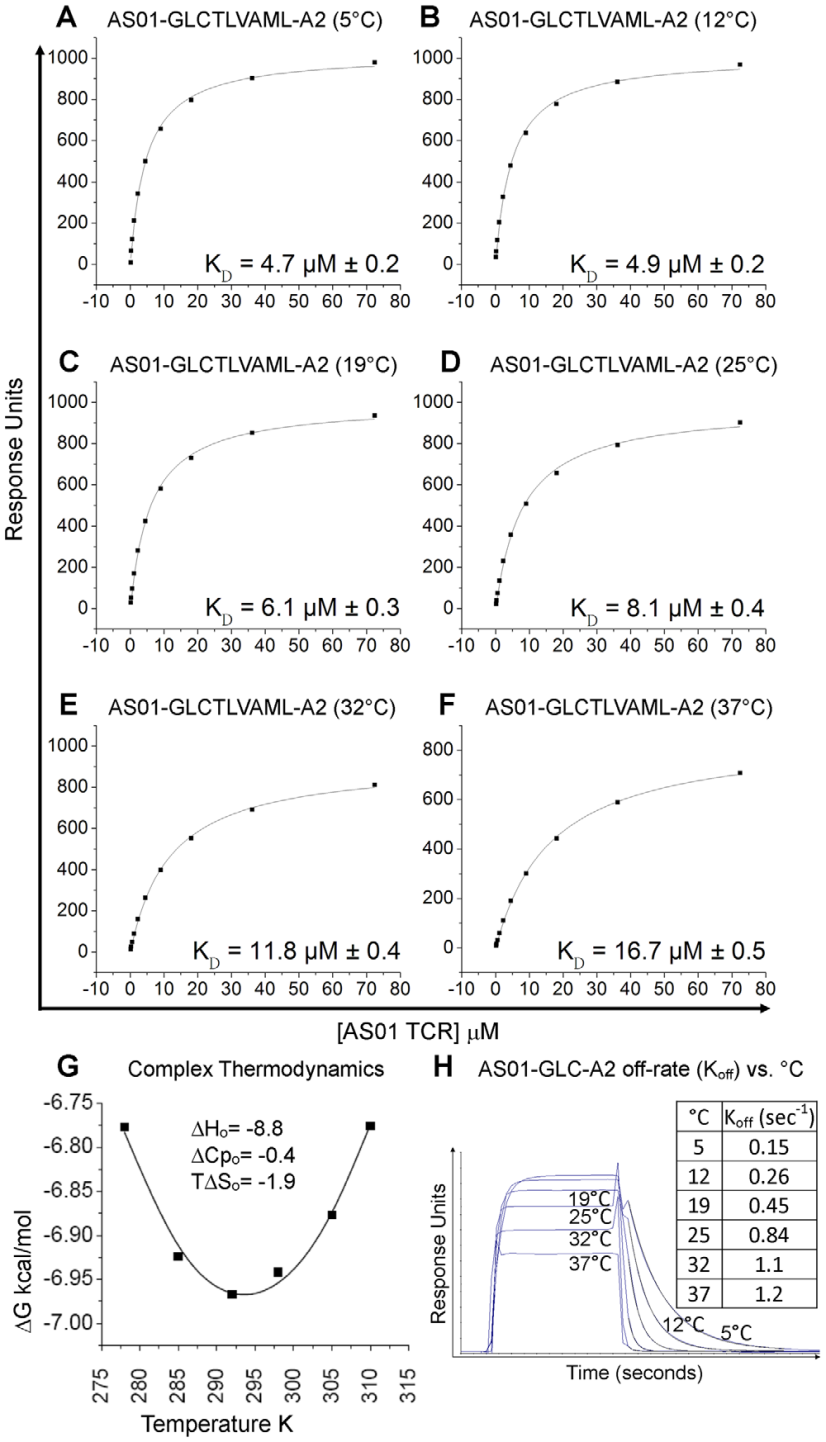
Binding affinity and thermodynamics of the AS01-GLC-A2 interaction. SPR measurements were conducted at different temperatures as shown. Ten serial dilutions of the AS01 TCR were measured in triplicate at each temperature; the mean response for each concentration is plotted. (A-F) The equilibrium binding constant (K_D_) values were calculated in each case using a nonlinear curve fit (*y* =  (P_1_ *x*)/P_2_ + *x*)); mean plus SD values are shown. (G) The thermodynamic parameters were calculated according to the Gibbs-Helmholtz equation (ΔG° = ΔH − TΔS°). The binding free energies, ΔG° (ΔG° = -RTlnK_D_), were plotted against temperature (K) using nonlinear regression to fit the three-parameter equation, (y = dH+dCp*(x-298)-x*dS-x*dCp*ln(x/298)), as previously reported [69]. (H) AS01 TCR (18.1 µM) was injected over GLC-A2 at 5, 12, 19, 25, 32 and 37°C. The responses observed with injections of AS01 TCR over a control sample were deducted. Off-rates (K_off_) were calculated assuming 1∶1 Langmuir binding using a global fit algorithm (BIAevaluate 3.1). Data and fits are shown.

To complement information gained from the crystal complex, we dissected in detail the affinity and thermodynamics of the public AS01 TCR. To achieve this, the binding strength of the AS01-GLC-A2 complex was measured at 5, 12, 19, 25 and 37°C using surface plasmon resonance (SPR). At 25°C, the K_D_ of the complex was 8.1 µM (Figure 3; Figure S1). This baseline affinity falls within the range of previously published human TCR/pMHC-I complexes (0.1–100 µM) and is typical of human TCR/pMHC-I complexes from viral systems (0.1–21 µM) [19], [24], [25] (Table 3). Interestingly, we observed that the affinity of the AS01-GLC-A2 complex interaction decreased with increasing temperatures; thus, K_D_ values gradually decreased from 4.7 µM at 5°C to 16.7 µM at 37°C (Figure 3A–G). This difference was mainly due to a much faster off-rate (K_off_) at higher temperatures (K_off_  = 1.2 sec^−1^ at 37°C compared to 0.15 sec^−1^ at 5°C) (Figure 3H). Thus, at physiological temperature (37°C), the AS01 TCR binds with weaker affinity than at the standard measurement temperature (25°C). This difference may impact the antigen sensitivity of CD8^+^ T cells bearing this public receptor *in vivo*.

The affinity of an interaction can be represented as its binding free energy, ΔG° (ΔG° = -RTlnK_D_). This binding energy is the sum of enthalpic (ΔH) and entropic (−TΔS) components as calculated using the Gibbs-Helmholtz equation (ΔG° = ΔH − TΔS°), either of which can be favourable (act to increase the affinity) or unfavourable (act to decrease the affinity). The binding of proteins is also accompanied by a change in the heat capacity ΔCp°. In order to calculate ΔH°, TΔS° and ΔCp°, the binding constant data were subjected to van't Hoff analysis by plotting the binding ΔG° versus temperature (K) using nonlinear regression to fit the three-parameter equation to the curve (see Materials and Methods). The AS01-GLC-A2 interaction was characterized by a binding ΔG° of −6.9 kcal/mol at 25°C (the standard for measuring TCR/pMHC parameters [26]), which is within the normal range for TCR/pMHC interactions [27]. The energy of the interaction was probably derived primarily from a net increase in the formation of new noncovalent bonds (hydrogen bonds, salt bridges and van der Waals contacts) during complex formation, evident from the favorable enthalpy (ΔH° = −8.8 kcal/mol). Notably, this TCR/pMHC interaction is entropically unfavourable (TΔS° = −1.9 kcal/mol), although this value lies at the lower end of the scale of published TCR/pMHC entropic values (−0.4 to −29 kcal/mol) [27]. The relatively small ΔCp° value of −0.4 kcal/mol·K is within the range of other TCR/pMHC complexes [27] (Figure 3G), which conforms with the normal SC value for this complex. Next, we investigated the thermodynamic properties of the AS01-GLC-A2 complex at the physiologically relevant temperature of 37°C. At this temperature, the binding free energy (ΔG° = −6.8 kcal/mol) was very similar to the binding energy observed at 25°C (ΔG° = −6.9 kcal/mol). However, at 37°C, the interaction was driven strongly by enthalpy, evident from the decrease in ΔH° to −14.1 kcal/mol (37°C) (Figure S3). This resulted in a larger entropic cost to complex formation (TΔS° = −7.3 kcal/mol). Thus, at 37°C, the interaction between the AS01 TCR and GLC-A2 is more enthalpically driven, with a greater entropic penalty, compared to 25°C.

### Verification of TCR binding hot spots

**Figure 4:**
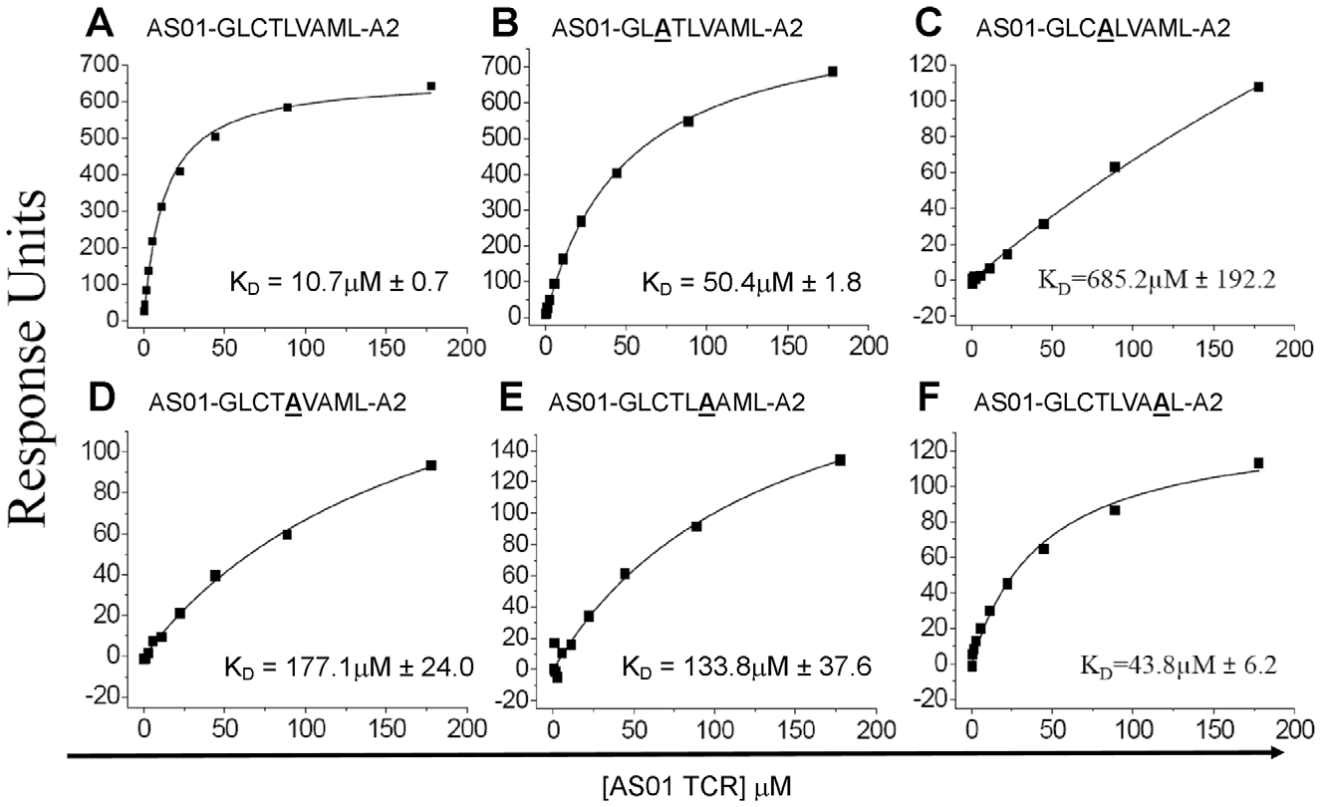
Binding affinities of the AS01 TCR with GLC-A2 variants. Equilibrium binding analysis at 25°C for wildtype GLC peptide (A) and alanine mutants (B-F). Ten serial dilutions of the AS01 TCR were measured in triplicate for each equilibrium experiment; the mean response for each concentration is plotted. The equilibrium binding constant (K_D_) values were calculated using a nonlinear curve fit (*y* =  (P_1_ *x*)/P_2_ + *x*)) as previously reported [24], [26], [37]; mean plus SD values are shown.

The AS01-GLC-A2 structure indicated that Thr4, Val6 and Met8 of the peptide are important for TCR docking. Indeed, the AS01 TCR contacts only these residues in the peptide. To verify the importance of these contact areas, we performed an Ala mutagenesis scan across the peptide backbone and evaluated the capacity of the pMHC-I mutants to bind the AS01 TCR using SPR (Figure 4; Figure S2). As peptide positions P1, P2 and P9 are often buried and/or important for MHC binding, we focused on assessing the solvent exposed positions P3, P4, P5, P6 and P8. We did not assess P7, as this residue is Ala in the native sequence. As expected, given that no contacts were made with Cys3, mutation of P3 had a relatively small effect on TCR binding (∼5-fold reduction) (Figure 4). Conversely, mutation of Thr4 reduced AS01 TCR binding by more than 60-fold to 685.2 µM, presumably through loss of two side-chain contacts provided by the CDR1α and CDR3α loops. Interestingly, mutation at Leu5 resulted in a ∼17-fold reduction in affinity to 177 µM. While Leu5 is not a TCR contact residue, it does affect the total peptide backbone structure by providing a secondary anchor that results in a kink in the centre of the peptide backbone. This kink, made via hydrogen bonds with main chain atoms of Val152 and Leu156 of the MHC α-1 helix, pulls Leu5 into the MHC groove. In this conformation, Thr4 and Val6 form two “humps” on either side of Leu5. The mutation of Leu5 to Ala would probably result in the loss of this MHC anchoring and allow the peptide backbone to relax in the cleft. This backbone loosening would likely push Thr4 and Val6 into new conformations, resulting in the loss of original contacts. As expected, mutation of TCR contact residue Val6 resulted in a considerable reduction in affinity to 133.8 µM (∼13-fold). This affinity reduction is likely caused by the loss of two non-polar interactions between the Val6 side-chain atoms and the CDR3β loop. Finally, mutation of Met8 reduced the affinity of AS01 TCR binding to 43.9 µM. This modest effect can likely be explained by the loss of a single, side-chain-derived van der Waals bond with the CDR1β loop. The original hydrogen bond, formed by the CDR3β and the main-chain atoms of Met8, would likely remain when Met8 is mutated to Ala.

### Surface complementarity (SC) preference of the AS01 TCR

The SC program, from the CCP4 suit [28], is able to index the binding potential of two molecules between 0.0 (no SC) and 1.0 (perfect SC). As well as overall SC, SC indices can be partitioned to different zones of the contact face. This can help specify where two molecules invest the bulk of their contact energies. We calculated the SC of the AS01 TCR between: (i) the whole GLC-A2 molecule; (ii) just the HLA A*0201 molecule; and, (iii) just the peptide (Table 3). We also extended this SC assessment to a full meta-analysis of all conventional human TCR/pMHC-I complexes solved to date (Table 3). Alloreactive structures were omitted from the meta-analysis. This review also included BSA and binding affinities of the complex set, as well as the number of contacts made between TCR and peptide within 3.4Å. The AS01-GLC-A2 complex exhibited a SC index of 0.640, which is average for TCR/MHC-I complexes (mean = 0.648). Interestingly, while the total pMHC-I SC appeared typical, the AS01 TCR revealed a SC preference for MHC-I (SC = 0.676) over peptide (SC = 0.577). The large majority of TCR/MHC-I complexes studied thus far reliably exhibit a higher SC index for peptide compared with MHC. In fact, only the JM22 TCR joins AS01 in this unusual, large-scale switch of region preference.

### Convergent recombination potential of the AS01 TCR

**Figure 5:**
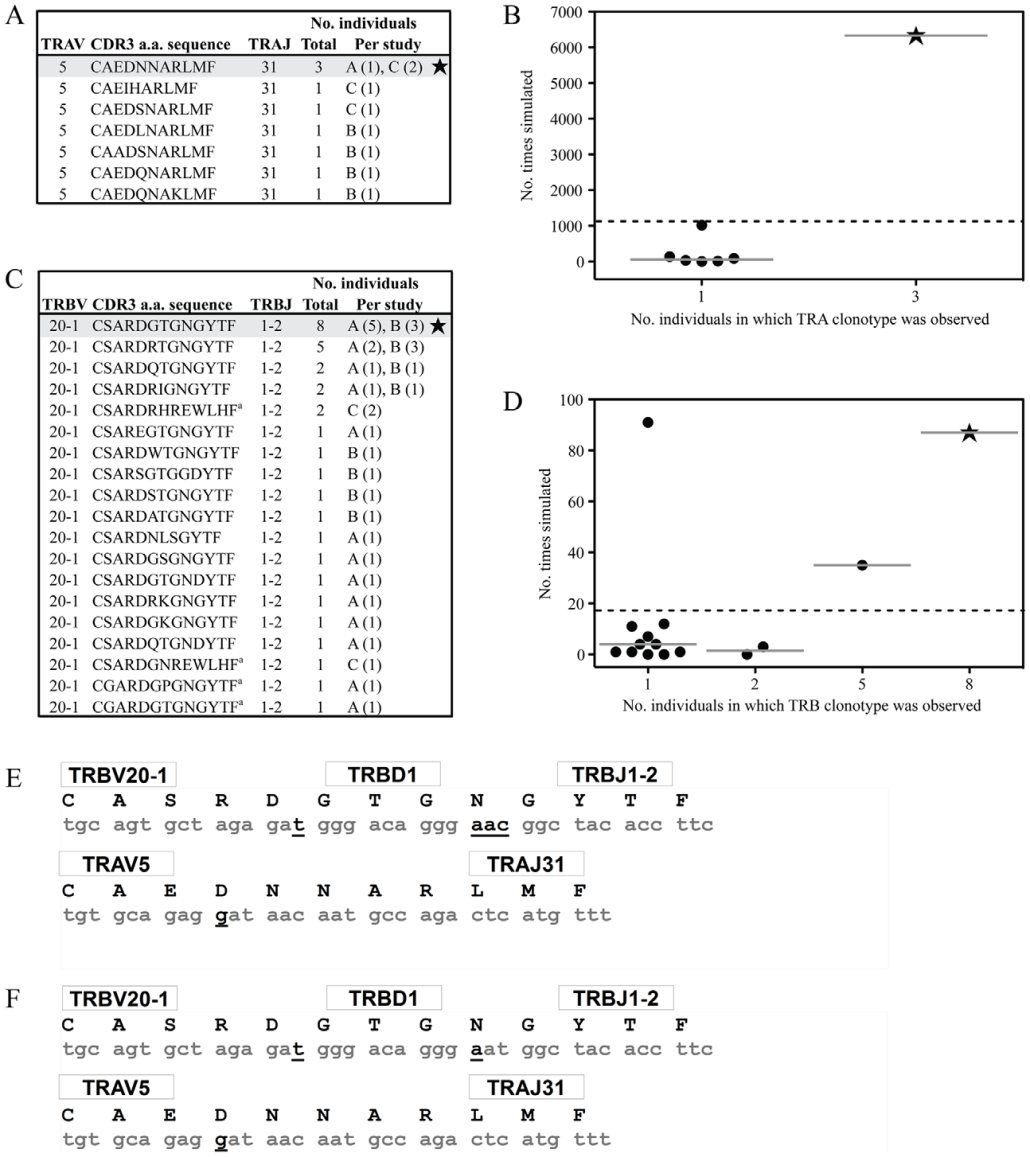
Convergent recombination analysis of the AS01 TCR. Previously reported TRAV5/TRAJ31 (A) and TRBV20-1/TRBJ1-2 (C) amino acid clonotypes specific for the GLC-A2 epitope are listed together with the number of individuals in which each clonotype was observed. Sequences were mined from previous studies A [8], [17], B [7] and C [6]. To assess the role of convergent recombination and TCR production frequency in the inter-individual sharing of these TRAV5/TRAJ31 and TRBV20-1/TRBJ1-2 amino acid clonotypes, computer simulations of a random V(D)J recombination process involving either the TRAV5/TRAJ31 or TRBV20-1/TRBJ1-2 gene combinations were used to estimate their relative production frequencies. The number of times that each observed GLC-specific TCR amino acid sequence was generated in the simulations of a random V(D)J recombination process is shown for the TRAV5/TRAJ31 (B) and TRBV20-1/TRBJ1-2 (D) amino acid clonotypes versus the number of individuals in which each sequence was observed. The solid horizontal lines represent the medians of the number of times that TCR sequences observed in a particular number of individuals were simulated. The dashed horizontal lines extending the width of the plots represent the mean frequency of sequence generation, across all observed GLC-specific TRAV5/TRAJ31 or TRBV20-1/TRBJ1-2 amino acid clonotypes, regardless of the number of individuals in which they were found. The data shown for the TRBV20-1/TRBJ1-2 clonotypes have been published previously [17] and are shown here for completeness. ^a^ Four TRB clonotypes could not be made with the parameters used in the simulation; these are not included in panel D. The most shared GLC-specific TCRα amino acid clonotype (TRAV5/CAEDNNARLMF/TRAJ31) was encoded by 18 different nucleotide sequences and produced by a total of 117 different recombination mechanisms (i.e. different splicings of the germline genes and nucleotide additions) in the simulations. The most shared GLC-specific TCRβ amino acid clonotype (TRBV20-1/CSARDGTGNGYTF/TRBJ1-2) was encoded by 16 different nucleotide sequences and produced by a total of 47 different recombination mechanisms. The CDR3 nucleotide sequences coding for the AS01 TCR (E) and the CDR3 nucleotide sequences coding for the most shared TCRα and TCRβ amino acid clonotypes in the GLC-specific response (F). The corresponding TRBV, TRAV, TRBD, TRBJ and TRAJ genes are listed. Germline-derived nucleotides are highlighted in grey and non-germline-derived nucleotides are bolded and underlined.

Along with obvious structural features that promote AS01 selection, underlying genetic factors are likely to exist that elevate the frequency at which this receptor is manufactured. We have previously identified a process of convergent recombination that may enable some TCRs to be produced more efficiently than others [29]. Using computer simulations of a random V(D)J recombination process we have demonstrated for numerous systems [17], [30], [31] that convergent recombination leads to large differences in TCR production frequencies, even in the case of completely unbiased gene recombination. These simulations account for the various mechanisms that contribute to the production of TCR nucleotide and amino acid sequences, including *TRV*, *TRD* and *TRJ* gene splicing, N nucleotide additions, recurrent CDR3 motifs and codon redundancy. We have previously shown that the public TCR β-chain (TRBV20-1/CSARDGTGNGYTF/TRBJ1-2) is the most common chain used *in vivo* in the GLC-specific response and is the second most frequent GLC-A2-specific TRBV20-1/TRBJ1-2 chain made *in silico* by random gene recombination [17]. We have expanded this TCR β-chain analysis to include additional published clonotypes [6], [7] and confirm our previous results here (Figure 5C–D). In addition, we simulated the production of TCR α-chains using the TRAV5 and TRAJ31 genes to assess the relative production frequencies of GLC-A2-specific TCR α-chain sequences based on previously identified *in vivo* clonotypes [6], [7]. The simulation indicated that the public TCR α-chain (TRAV5/CAEDNNARLMF/TRAJ31) is the most efficiently produced TRAV5/TRAJ31 combination in the GLC-A2 response (Figure 5A–B), being generated ∼6 times more frequently *in silico* than the next most efficiently generated clonotype CAEIHARLMF. Aiding the ease of production, both the public TCR α- and β-chain amino acid sequences can be encoded by nucleotide sequences requiring few N nucleotide insertions The AS01 β-chain contains just 4 N nucleotide insertions, with only Asn103 being randomly encoded (Figure 5E). Furthermore, the AS01 TCR β-chain can been observed *in vivo* to be manufactured with just two N nucleotide additions (Figure 5F) [17]. The AS01 TCR α-chain contains just a single N nucleotide insertion, partially encoding Asp93.

## Discussion

The archetypal AS01 TCR is likely to be one of the most common and numerically frequent αβ TCRs in humans. This is based on the following considerations: (i) the HLA-A*0201 allele is arguably the most common and widespread MHC-I allele in humans, with frequencies above 60% in certain regions [32]; (ii) EBV is one of the most successfully disseminated human pathogens, persistently infecting more than 90% of individuals [33]; (iii) the GLC-A2 antigen is one of the most immunodominant CD8^+^ T cell targets across the EBV proteome [4]; (iv) GLC-specific CD8^+^ T cell responses are amongst the largest observed, both in the EBV system and in comparative terms with respect to other human pathogens studied thus far [4]; and, (v) the GLC-specific response is dominated by CD8^+^ T cells that bear a public TRAV5/TRBV20-1 receptor [17].

Structurally, most features exhibited by the AS01 TCR are within the parameters previously seen in the TCR/pMHC-I system [19]. AS01 docks in a roughly diagonal fashion to the pMHC and is positioned centrally above the peptide. The TCR α-chain is positioned above the peptide N-terminus and the TCR β-chain is positioned above peptide C-terminus. The peptide is engaged chiefly by the CDR3 loops with additional support from the CDR1 loops. AS01 interacts with the MHC helices via residues within the CDR1 and CDR2 loops, which include bonds with universal MHC anchors Arg65 and Gln155. Gln155 is proposed to be a “gatekeeper” residue guiding MHC-I-restricted TCR recognition as it is universally contacted by all αβ TCRs studied to date and often switches conformation between bound and unbound forms [23], [34]. Interestingly, however, the AS01 TCR does not contact Arg69, which represents the third member of the classical MHC “restriction triad” [34]. Biophysically, the AS01 TCR binds GLC-A2 with a K_D_ of 8.1 µM, which is typical for TCR interactions with viral MHC-I-restricted antigens [19], [24]. The AS01-GLC-A2 complex also has an average BSA (of 2134 Å^2^) that is within, if not towards the higher end, of the TCR/pMHC system. One notable structural trait of the AS01 TCR is its SC preference. The large majority of TCR/pMHC-I complexes have a peptide>MHC SC bias. This bias is inverted in the AS01-GLC-A2 complex. Again, interestingly, only the JM22 TCR, which is also public and HLA-A*02-restricted, exhibits this inverted preference. Ultimately though, this particular trait cannot be exclusively assigned to public TCRs since the RA15 and LC13 TCRs show conventional SC preference.

Thermodynamic analysis revealed that the interaction between the AS01 TCR and GLC-A2 was within the normal range of other reported TCR/pMHC interactions [27]. Importantly, the interaction was strongly enthalpically driven; thus, stabilization of the TCR/pMHC interface through the formation of noncovalent bonds is likely to be the chief factor driving complex formation. In addition, the complex exhibited unfavourable entropy, indicating that there was a net gain of order during TCR/pMHC binding. This observation indicates that the entropic energy generated by expulsion of solvent (ordered water molecules) upon complex formation is countered by the entropic energy penalty attributed to the conformational ordering of the TCR CDR loops and pMHC surface during docking. These data are in agreement with previous thermodynamic analyses of TCR/pMHC interactions, which show that the favorable enthalpic energy generated by the formation of a relatively large number of new contacts at the interface is countered by a large unfavorable entropic cost that results in a relatively weak binding affinity compared to other protein-protein interactions [27], [35], [36], [37]. Lastly, we investigated the thermodynamic properties of the AS01-GLC-A2 complex at the physiologically relevant temperature of 37°C. Notably, at 37°C, the interaction between the AS01 TCR and GLC-A2 was more strongly enthalpically driven, with a greater entropic penalty, compared to 25°C. Importantly, these differences in binding energy almost halved the affinity of AS01 TCR binding at 37°C compared to the standard measurement temperature of 25°C. Kinetic analysis revealed that the reduction in binding affinity was primarily attributed to a much faster off-rate at higher temperatures (Figure 3H). Thus, the AS01 TCR appears to bind less optimally to GLC-A2 at physiological temperatures (37°C) compared with the standard temperature used for SPR measurements *in vitro* (25°C).

The mutagenesis scan across the GLC peptide highlighted a number of critical zones for TCR recognition. As expected, mutation of the prominent peptide hump residues, Thr4 and Val6, resulted in 10–70 fold loss in affinity. A relatively surprising finding was the 17-fold loss in affinity following mutation of Leu5. Leu5 is not involved in TCR contacts and points down into the MHC cleft. Leu5 does, however, act as a secondary anchor for the GLC peptide, reinforcing the peptide backbone. Loss of this reinforcement would likely result in the peptide backbone ‘sinking’ into the MHC cleft, producing a less interactive interface. Recently seen in other systems [38], this observation highlights the importance of peripheral residues in TCR engagement. A note of broader interest is that every mutation along the GLC peptide resulted in a reduction of affinity (40 µM and above), whether within a TCR contact zone or not. TCRs specific for class-I-bound antigens of viral origin typically operate in the K_D_ <10 µM range and none have been seen over 30 µM [24]. Thus, a mutation at any of these points could result in suboptimal engagement with the archetypal public TCR. This would likely result in either the public TCR being outcompeted by higher affinity options *in vivo* or a hole in the TCR repertoire and possibly a reduction in immunogenicity. Interestingly, there is no evidence that EBV attempts to escape from the GLC response, as seen by complete epitope conservation across all known strains and isolates (GeneBank). This is remarkable given the considerable genetic variation between EBV strains [39], [40] and within some T cell epitopes [41], [42], [43], [44]. It is certainly conceivable that EBV has at least some flexibility to mutate the GLC backbone without a significant loss of viral fitness. Hence, a question presents itself. Why does EBV not try to escape from one of the most potent T cell responses raised against it? In assessing this question, it is important to note that genetic evidence from EBV studies suggests that evolutionary pressure on T cell epitopes is directed towards their conservation rather than their inactivation [45]. This leads to speculation that epitope conservation may be advantageous to the virus. Thus, some highly immunogenic epitopes could be maintained deliberately to elicit large fleets of CD8^+^ T cells, perhaps to regulate viral replication, minimize pathology and maintain a peaceful coexistence with the host. Alternatively, the large T cell responses generated by these epitopes may aid the virus as bonus replicative tissue. In addition to the well established tropic tissues, B cells and epithelia, EBV has recently been found in several human tissues including T cells [46]. A related question is whether TCR affinity influences epitope variation. The affinity of the AS01 TCR, at physiological temperature, is in the lower half of the range reported for anti-viral TCRs to date [24]. Could this small decrease in relative affinity place less selection pressure on the GLC epitope? This is an interesting question. The answer is likely influenced by the intrinsic mutagenic potential of the epitope.

Genetically, the public AS01 TCR is primarily assembled from chromosome-derived DNA (Figure 5E). The TCR β-chain and TCR α-chain incorporate just 4 and 1 non-germline nucleotide/s, respectively. Only one residue in the receptor (Asn103β) is encoded wholly by non-germline DNA. Interestingly, in many individuals, Asn103β in the AS01 TCR is majority encoded by the TRBJ1-2 gene (Figure 5F) [17]. Thus every residue in the AS01 TCR can be wholly or partially encoded by germline DNA. This “germline-rich” feature of the TCR α-chain and TCR β-chain amino acid sequences of AS01 contributes towards the prediction that these are frequently produced by convergent recombination [30]. The public AS01 TCR comprises the *TRBV20-1*, *TRBD1*, *TRBJ1-2*, *TRAV5* and *TRAJ31* genes. Structural analysis revealed that each of these *TR* genes encode unique residue patterns that appeared specialized for the GLC-A2 ligand. Critical docking residues, found within the TCR/pMHC interface, were exclusively encoded by the *TR* genes listed above and were not resident in the other 168 genes available on the TCR α and β loci. Thus, this specific structural architecture required to preserve the AS01 docking mode explains the TRBV, TRBJ, TRAV, and TRAJ bias in the GLC-A2 CD8^+^ T cell response.

The AS01-GLC-A2 complex necessitates comparison with other published public TCR structures including the LC13 TCR [47] and the JM22 TCR [48]. The LC13 TCR is specific for the FLRGRAYGL (FLR) peptide from EBV and restricted by the HLA-B*0801 molecule. The LC13 TCR, composed of *TRBV7-8/TRBJ2-7* and *TRAV26-2/TRAJ52* gene products, is residue-identical between individuals (Type III bias) [49], [50], [51]. Intriguingly, the LC13 TCR is also heavily comprised of germline DNA. The TCR β-chain can be constructed wholly from germline DNA [49], [50], [51]. The TCR α-chain shows a similar degree of germline composition and only a single residue (Pro93) is encoded by DNA of non-germline origin. The TCR/pMHC complex reveals that the LC13 TCR engages its cognate ligand at a crossing angle of 42° [47], virtually identical to that of AS01. Also akin to AS01, the contact footprint was evenly split between the TCR α- and β-chains. The LC13 TCR was seen to adjust conformation during ligation, manoeuvring its CDR3 loops around two central solvent expose residues at P6 and P7. The AS01 TCR also engaged central residues of the peptide; however, it is unknown whether AS01 also undergoes conformational change upon ligation, as this would require the TCR structure in an unligated state. A structural basis underlying the selection of LC13 TCR was evident upon examination of the complex. Contact residues exclusively encoded by the constituent genes were critical for specific engagement [47], and mutation of these germline-encoded residues abrogated recognition [50]. Overall, the LC13 and AS01 TCRs exhibit close genetic and structural parallels. However, the investment of germline DNA composition alternates between the receptors. Thus, the LC13 TCR exhibits more germline composition on the β-chain compared with the α-chain. This pattern is inverted in the AS01 TCR. It is also worth noting that the HLA-B*0801 allele, to which the LC13 TCR is restricted, is arguably the most common HLA-B allele in Caucasian populations. The JM22 TCR is specific for the GILGFVFTL (GIL) peptide from the influenza virus and is restricted by the HLA-A*0201 molecule. In this response, gene bias is skewed to *TRBV19* with a common Arg residue in the CDR3β loop [52], [53]. However, in contrast to the GLC- and FLR-responses, the GIL-specific repertoire is more variable and identical TCRs are not always apparent across individuals. A number of different *TRBJ* genes are used in the repertoire along with variation in CDR3β residue composition and CDR3β length [52], [53]. The paired TCR α-chain is also variable, with fluctuation in *TRAV* gene usage. In general, the GIL-specific response is an example of Type IV bias. The TCR/pMHC complex revealed that JM22 engages its cognate ligand at a crossing angle of 62° [48], which is more orthogonal compared with AS01. As suggested by the above mentioned gene bias, the contact footprint is considerably “β-centric”, with residues within the *TRBV19*-encoded CDR1 and CDR2 loops dominating pMHC engagement. This docking modality allowed the conserved Arg in the CDR3β loop to peg the TCR between the peptide and MHC groove. A detailed mutational analysis revealed that *germline*-encoded residues were critical for antigen specificity and, along with CDR3β Arg peg, it was hypothesized that the *TRBV19* gene may have been evolutionary useful during recurrent influenza pandemics [54].

The germline-rich composition of the AS01 TCR draws parallels with the TCRs displayed by iNKT cells from the innate immune compartment. Here, the iNKT TCR α-chain (TRAV10/TRAJ18) can be manufactured wholly by germline DNA [55]. Structural analysis of an iNKT TCR bound to its cognate CD1d-α-GalCer ligand revealed that the receptor docks in a parallel fashion to the antigen cleft [56], a docking modality very different to the diagonal docking of AS01 and the other MHC-restricted receptors [19]. In this parallel docking mode, the CDR1α and CDR3α loops were seen to dominate the contact footprint across both CD1d and the antigen; the residues involved in these contacts were exclusively encoded by the *TRAV10* and *TRAJ18* genes, providing a structural basis for invariant gene bias. A further mutational study across the iNKT TCR confirmed the critical importance of these germline-encoded CDR1α and CDR3α residues for ligand recognition [57]. These studies reinforce the observation that extreme biases within TCR repertoire formation are likely shaped through highly specific structural requirements of the target ligand. The iNKT TCR differs from the AS01 TCR in that it that has an unprecedentedly small BSA (of 910Å^2^), likely as a result of the α-chain binding towards the terminus of the binding cleft. This binding mode pushes the β-chain towards the extreme end of the binding cleft and limits its role during engagement [56], [58], providing a structural basis for the highly diverse nature of the iNKT β-chain repertoire *in vivo* [55]. Conversely, the AS01 TCR α- and β-chains are both public, and the contact footprint is more evenly spread across both chains.

EBV has engaged our species and its antecedents for approximately 80 million years [59]. During this entwined co-evolution, countless immune assaults and counter-assaults would have been waged, with many genes formed and lost. Natural selection and hereditary transmission would have progressively bestowed useful genes for host defence and virus offence. It is likely that the 450 million year old combinatorial immune system [60], and more specifically the highly polymorphic TCR and MHC gene set, would have been intimately involved in this “arms race”. Thus, it is conceivable that public TCRs, such as AS01, may be very old defence structures, easily formed and found on the chromosome, that provide a naive pre-emptive defence net against almost-certain infection by primordial pathogens. Conversely, it could be argued public TCRs are simple by-products of biases in the V(D)J recombination system, according to which some receptor combinations leave the thymus more often than others. In conflict with this straightforward genetic hypothesis is the observation that some public TCRs exist with the same amino acid CDR3 sequences that are redundantly encoded by largely non-germline derived DNA [49]. This indicates that, during competitive antigen-driven selection, there is some structural advantage already encoded in the original germline sequence. That is, we appear to be born with αβ TCR fragments already lying in the chromosome that are exquisitely specific for EBV targets. Given the time scale of this conflict, it is intriguing to consider whether public TCRs have evolved to aid the host or EBV itself; perhaps they could even represent some middle ground that favours a largely peaceful coexistence? Ultimately though, the role of evolution in guiding this phenomenon is unknown and can only be conjectured. After all, these *TRBV* and *TRAV* gene segments are likely useful in defence against other pathogens. In addition, TCR variable genes appear to have an innate preference for MHC [61], so the idea of a germline receptor having both highly tuned specificity and broad cross-reactive potential is, while not strictly mutually exclusive, an interesting observation. Aside from the origins of the public receptor phenomenon, the AS01-GLC-A2 complex may provide a fascinating glimpse of an ancient immune battle, fought quietly and in the same way, possibly billions of times across the two hundred thousand years of *homo sapiens* history [62].

## Materials and Methods

### T cell clone generation and TCR isolation

The AS01 CD8^+^ T cell clone was generated from a healthy EBV^+^, HLA-A*0201^+^ individual as described previously [63]. Briefly, PBMC were stimulated with 1 µM GLCTLVAML peptide and cloned via limiting dilution. The AS01 TCR was identified as described previously [64]. Briefly, total RNA was extracted from 10^5^ T cells using TRIzol reagent and a RT-PCR was performed using Superscript III (Invitrogen Life Technologies). PCR was performed using a panel of TRAV- and TRBV-specific primers and the product was cloned into the pGEM-T vector system (Promega). The TCR product was sequenced using the ABI PRISM Big Dye termination reaction kit (Applied Biosystems) and the sequences were defined according to the international ImMunoGeneTics database (IMGT) TCR gene nomenclature [65].

### Generation of expression plasmids

The HLA-A*0201 (A2) α-chain and β2m sequences were generated by PCR mutagenesis (Stratagene) and PCR cloning. All sequences were confirmed by automated DNA sequencing. A disulphide-linked construct was used to produce the soluble domains (variable and constant) for both the TCR α- and β-chains [66], [67]. The soluble A2 α-chain (α-1, α-2 and α-3 domains) was tagged with a biotinylation sequence. All 4 constructs, TCRα, TCRβ, A2-tagged and β2m, were inserted into separate pGMT7 expression plasmids under the control of the T7 promoter [66].

### Protein expression, refolding and purification

Competent Rosetta DE3 *E.coli* cells were used to express the TCRα, TCRβ, A2-tagged and β2m proteins in the form of inclusion bodies (IBs) as described previously [66]. For a 1L TCR refold, 30 mg of AS01 α-chain IBs were incubated at 37°C for 15 mins with 10 mM DTT and added to cold refold buffer (50 mM TRIS pH 8.1, 2 mM EDTA, 2.5 M urea, 6 mM cysteamine hydrochloride and 4 mM cystamine). After 15 mins, 30 mg of AS01 β-chain, incubated for 15 mins at 37°C with 10 mM DTT, was added. For a 1 L GLC-A2 refold, 30 mg of A2 α-chain was mixed with 30 mg of β2m and 4 mg of the GLCTLVAML peptide (or GLC mutants) for 15 mins at 37°C with 10 mM DTT. This mixture was then added to cold refold buffer (50 mM TRIS pH 8, 2 mM EDTA, 400 mM L-arginine, 6 mM cysteamine hydrochloride and 4 mM cystamine). Refolds were mixed at 4°C for 1 hr. Dialysis was carried out against 10 mM TRIS pH 8.1 until the conductivity of the refolds was under 2 mS/cm. The refolds were then filtered and purified. Primary purification was conducted using an ion exchange (Poros50HQTM) column and secondary purification was conducted using a gel filtration (Superdex200HRTM) column. The protein was purified using either BIAcore buffer (10 mM HEPES pH 7.4, 150 mM NaCl, 3 mM EDTA and 0.005% (v/v) Surfactant P20) or crystallization buffer (10 mM TRIS pH 8.1, 10 mM NaCl). Protein quality was analyzed by Coomassie-stained SDS-PAGE.

### SPR analysis

Binding analysis was performed independently using a BIAcore 3000 and a BIAcore T100 equipped with a CM5 sensor chip as reported previously [68]. Between 200 and 400 response units (RUs) of biotinylated pMHC was immobilized to streptavidin, which was chemically linked to the chip surface. The pMHC was injected at a slow flow rate (10 µl/min) to ensure uniform distribution on the chip surface. Combined with the small amount of pMHC bound to the chip surface, this reduced the likelihood of off-rate limiting mass transfer effects. AS01 TCR was concentrated to 100 µM on the same day of SPR analysis to reduce the likelihood of TCR aggregation affecting the results. For equilibrium and kinetic analysis, ten serial dilutions were carefully prepared in triplicate for each sample and injected over the relevant sensor chips at 25°C. AS01 was injected over the chip surface using kinetic injections at a flow rate of 45 µl/min. For thermodynamic experiments, this method was repeated at the following temperatures: 12°C, 19°C, 25°C, 32°C, and 37°C. Results were analyzed using BIAevaluation 3.1, Microsoft Excel and Origin 6.1. The equilibrium binding constant (K_D_) values were calculated using a nonlinear curve fit (*y* =  (P_1_ *x*)/(P_2_ + *x*)). The thermodynamic parameters were calculated according to the Gibbs-Helmholtz equation (ΔG° = ΔH − TΔS°). The binding free energies, ΔG° (ΔG° = -RTlnK_D_) were plotted against temperature (K) using nonlinear regression to fit the three-parameter equation, (y = dH+dCp*(x-298)-x*dS-x*dCp*ln(x/298)), as reported previously [69].

### Simulation of V(D)J recombination

The process of convergent recombination encompasses the production of an amino acid sequence by a variety of nucleotide sequences and the production of a nucleotide sequence by a variety of recombination mechanisms (i.e. different germline gene contributions and nucleotide additions) [17], [29], [30], [31]. It also accounts for the frequent occurrence of some V(D)J recombination events due to the involvement of fewer nucleotide additions. To quantitatively assess the collective contribution of these various elements of convergent recombination in enhancing the production frequency of some TCR amino acid clonotypes relative to others in the absence of recombination biases, we used computer simulations of a random V(D)J recombination process [30] to estimate the relative production frequencies of the observed GLC-specific TCR amino acid clonotypes. The maximum number of nucleotide deletions from the ends of the TCR genes and the maximum number of nucleotide additions considered in the simulations were chosen to allow for the production of the majority of observed TCR sequences. For the TRAV5/TRAJ31 clonotypes, the simulated VJ recombination process allowed up to 10 nucleotide deletions from the 3′ end of the *TRAV5* gene, up to 16 deletions from the 5′ end of the *TRAJ31* gene, and up to 16 nucleotide additions. For each simulated TCR sequence, the number of nucleotide deletions from the 3′ end of the *TRAV5* gene, the number of nucleotide deletions from the 5′ end of the *TRAJ31* gene, and the number of nucleotide additions were determined from uniform distributions of the numbers of nucleotide deletions and additions. The nucleotide base of each of the nucleotide additions was randomly chosen. A total of 10 million in-frame TRAV5/TRAJ31 sequences were simulated. The computer simulations were performed using Matlab 7.9.0 (The Mathworks, Natick, MA).

### Crystallization, diffraction data collection and model refinement

AS01-GLC-A2 crystals were grown at 18°C by vapour diffusion via the hanging drop technique. 200 nL of 1∶1 molar ratio TCR and pMHC-I (at 10 mg/ml) was added to 200 nL of reservoir solution. Optimal crystals were obtained with 0.16 M calcium acetate hydrate, 0.08 M sodium cacodylate pH 6.0, 12.5% polyethylene glycol (PEG) 8000 and 20% glycerol. Data were collected at 100 K on beamline IO3 at the Diamond Light Source (DLS), Oxfordshire, UK. The AS01-GLC-A2 complex dataset was collected at a wavelength of 0.976Å using an ADSC Q315 CCD detector. Reflection intensities were estimated with the MOSFLM package [70] and the data were scaled, reduced and analyzed with SCALA and the CCP4 package [28]. The structure was solved with Molecular Replacement using AMORE [71] . The model sequence was adjusted with COOT [72] and the model refined with REFMAC5 [73]. Graphical representations were prepared with PYMOL [74]. Data reduction and refinement statistics are shown in Table 1. The reflection data and final model coordinates were deposited with the PDB database, assigned accession code 3O4L.

## Supporting Information

Figure S1 Panels A-F show raw data for the equilibrium binding analysis of the A2-GLC specific AS01 TCR at 5, 12, 19, 25, 32 and 37°C. The AS01 TCR was injected over a CM5 sensor chip with a negative control ligand immobilized on flow cell 1 (HLA-A*0201 in complex with ALAAAAAAV peptide) and the ligand under investigation immobilized on flow cell 2 (HLA-A*0201 in complex with GLCTLVAML peptide). The response unit increase observed during injection of the AS01 TCR over the control surface on flow cell 1 can be observed as the smaller response unit increase for each injection. The AS01 TCR was injected at the following concentrations; 0.1, 0.1, 0.3, 0.6, 1.1, 2.4, 4.5, 9.1, 18.1, 36.2 and 72.4 µM. The increase in the concentration of these injections can be observed by a larger increase in response units for each injection from left to right. All data were performed in triplicate. Representative data are shown.
(0.34 MB TIF)

Figure S2 Panels A-F show raw data for the equilibrium binding analysis of the A2-GLC specific AS01 TCR to the alanine substituted peptide variants. The AS01 TCR was injected over a CM5 sensor chip with a negative control ligand immobilized on flow cell 1 (HLA-A*0201 in complex with ALAAAAAAV peptide) and the ligand under investigation immobilized on flow cell 2 (HLA-A*0201 in complex with GLCTLVAML peptide, or an alanine substituted variant). The response unit increase observed during injection of the AS01 TCR over the control surface on flow cell 1 can be observed as the smaller response unit increase for each injection. The AS01 TCR was injected at the following concentrations; 0.3, 0.3, 0.7, 1.4, 2.8, 5.6, 11.1, 22.2, 44.5, 88.9 and 177.8 µM. The increase in the concentration of these injections can be observed by a larger increase in response units for each injection from left to right. All data were performed in triplicate. Representative data are shown.
(0.33 MB TIF)

Figure S3 The thermodynamic parameters of the A2-GLC specific AS01 TCR at 37°C were calculated according to the Gibbs-Helmholtz equation (ΔG°  =  ΔH − TΔS°). The binding free energies, ΔG° (ΔG°  =  -RTlnKD), were plotted against temperature (K) using nonlinear regression to fit the three-parameter equation, (y = dH+dCp*(x-298)-x*dS-x*dCp*ln(x/298)), as previously reported [24].
(0.79 MB TIF)
