# Supplementary figures and images for: Genetic and Structural Basis for Selection of a Ubiquitous T Cell Receptor Deployed in Epstein-Barr Virus Infection
Source: PLoS Pathog. 2010 Nov 18;6(11):e1001198. doi: 10.1371/journal.ppat.1001198 (PMC2987824; doi:10.1371/journal.ppat.1001198)

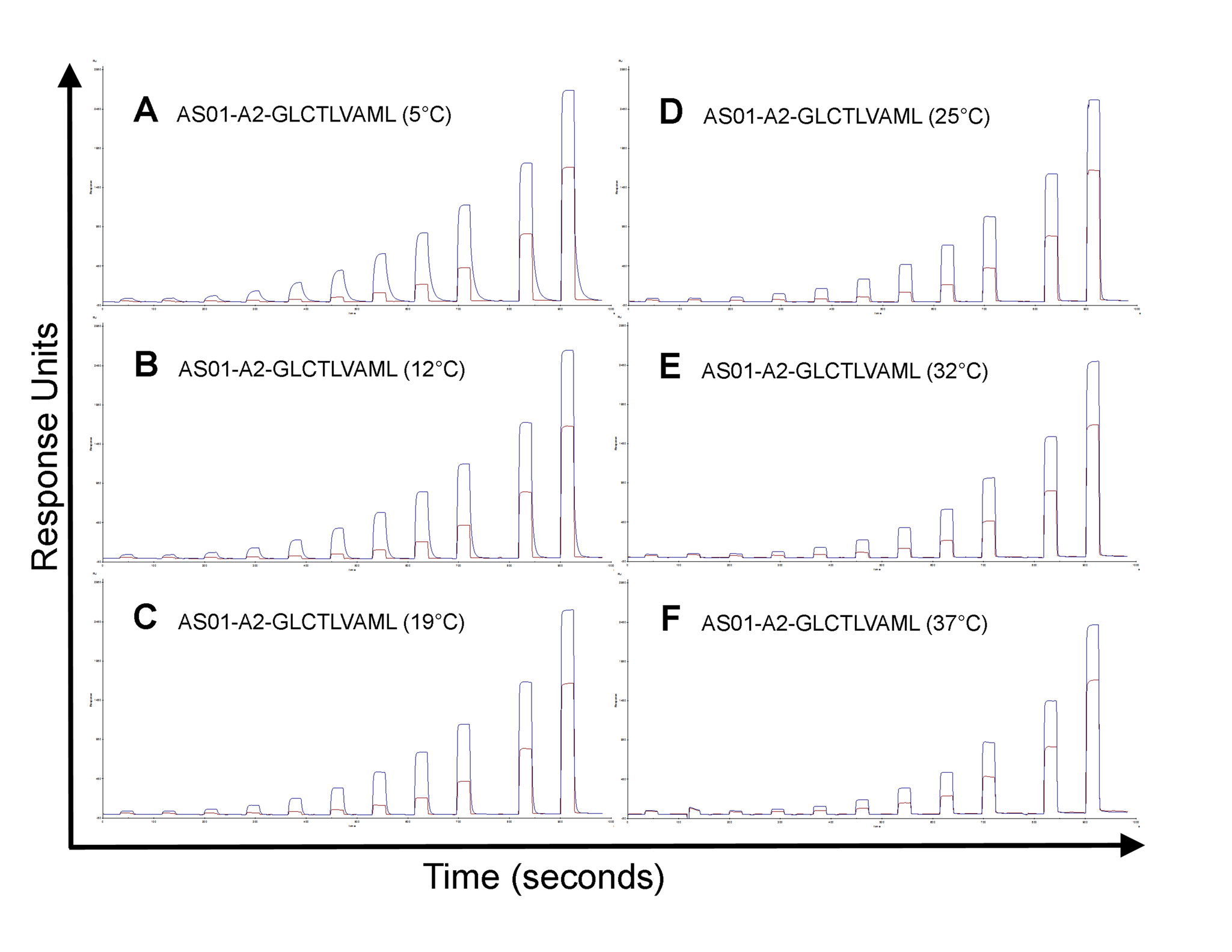

Supplement: Figure S1 — Panels A-F show raw data for the equilibrium binding analysis of the A2-GLC specific AS01 TCR at 5, 12, 19, 25, 32 and 37°C. The AS01 TCR was injected over a CM5 sensor chip with a negative control ligand immobilized on flow cell 1 (HLA-A*0201 in complex with ALAAAAAAV peptide) and the ligand under investigation immobilized on flow cell 2 (HLA-A*0201 in complex with GLCTLVAML peptide). The response unit increase observed during injection of the AS01 TCR over the control surface on flow cell 1 can be observed as the smaller response unit increase for each injection. The AS01 TCR was injected at the following concentrations; 0.1, 0.1, 0.3, 0.6, 1.1, 2.4, 4.5, 9.1, 18.1, 36.2 and 72.4 µM. The increase in the concentration of these injections can be observed by a larger increase in response units for each injection from left to right. All data were performed in triplicate. Representative data are shown. (0.34 MB TIF) [file ppat.1001198.s001.tif]

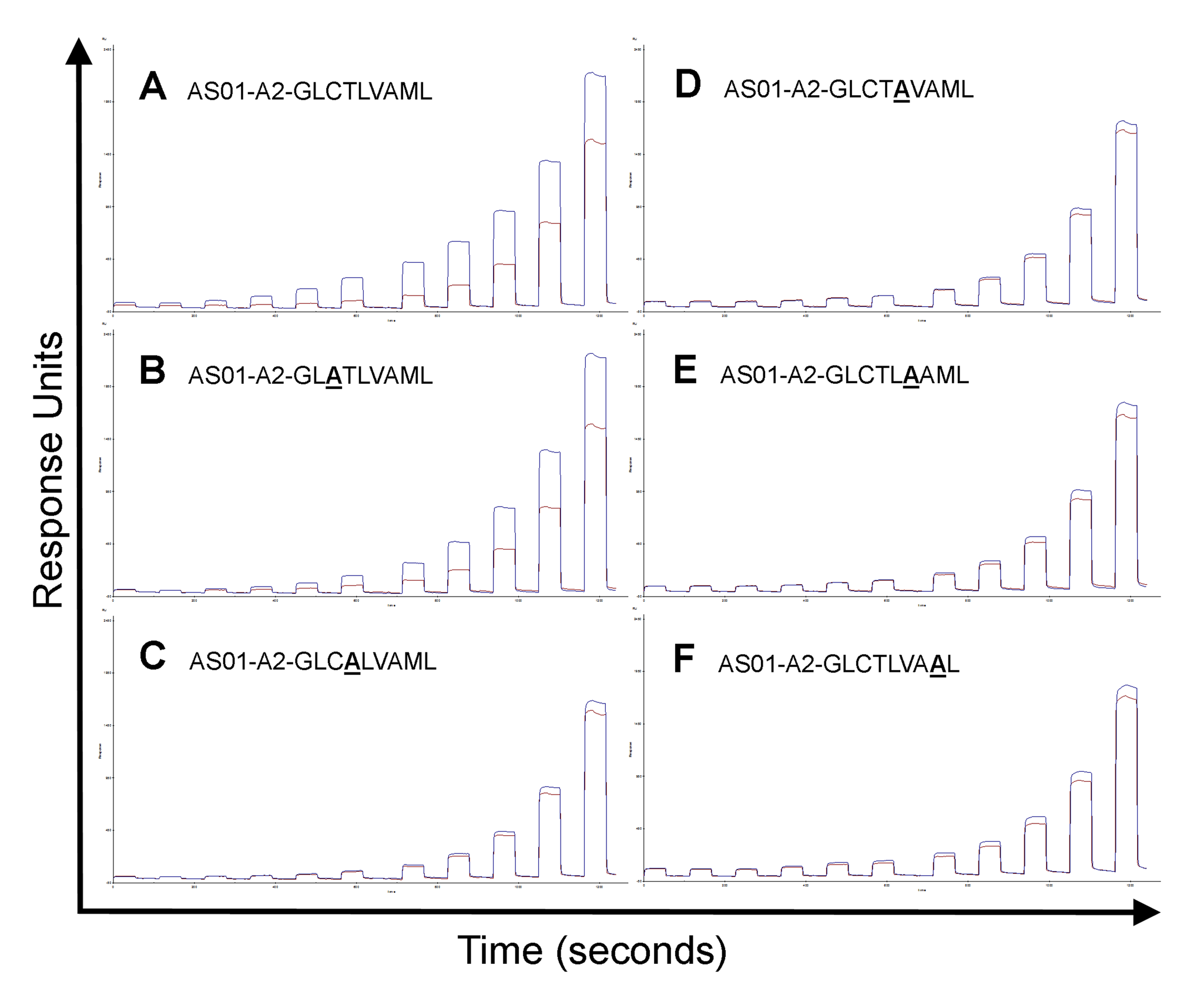

Supplement: Figure S2 — Panels A-F show raw data for the equilibrium binding analysis of the A2-GLC specific AS01 TCR to the alanine substituted peptide variants. The AS01 TCR was injected over a CM5 sensor chip with a negative control ligand immobilized on flow cell 1 (HLA-A*0201 in complex with ALAAAAAAV peptide) and the ligand under investigation immobilized on flow cell 2 (HLA-A*0201 in complex with GLCTLVAML peptide, or an alanine substituted variant). The response unit increase observed during injection of the AS01 TCR over the control surface on flow cell 1 can be observed as the smaller response unit increase for each injection. The AS01 TCR was injected at the following concentrations; 0.3, 0.3, 0.7, 1.4, 2.8, 5.6, 11.1, 22.2, 44.5, 88.9 and 177.8 µM. The increase in the concentration of these injections can be observed by a larger increase in response units for each injection from left to right. All data were performed in triplicate. Representative data are shown. (0.33 MB TIF) [file ppat.1001198.s002.tif]

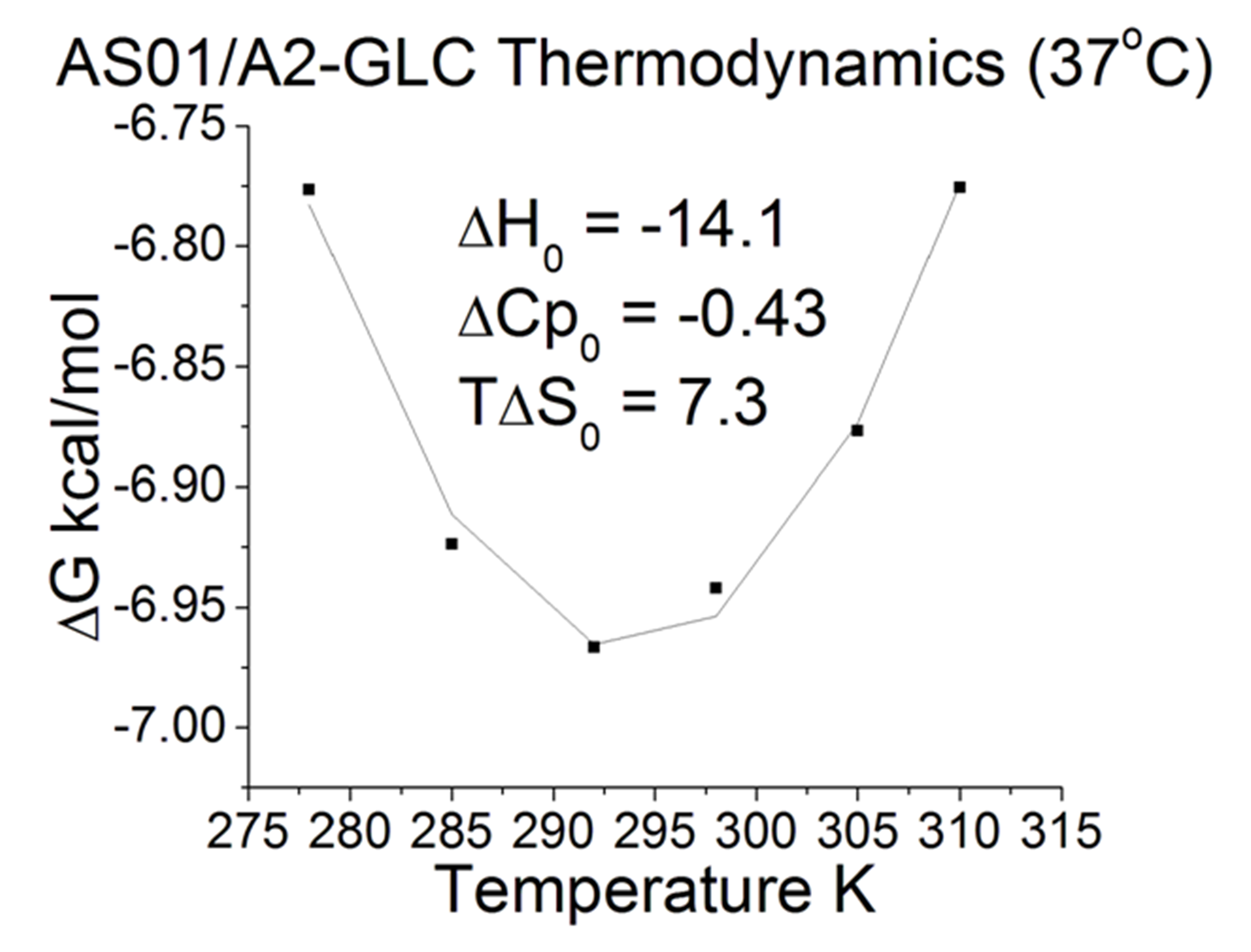

Supplement: Figure S3 — The thermodynamic parameters of the A2-GLC specific AS01 TCR at 37°C were calculated according to the Gibbs-Helmholtz equation (ΔG° = ΔH − TΔS°). The binding free energies, ΔG° (ΔG° = -RTlnKD), were plotted against temperature (K) using nonlinear regression to fit the three-parameter equation, (y = dH+dCp*(x-298)-x*dS-x*dCp*ln(x/298)), as previously reported [24]. (0.79 MB TIF) [file ppat.1001198.s003.tif]
